# The transport of nifurtimox, an anti-trypanosomal drug, in an *in vitro* model of the human blood–brain barrier: Evidence for involvement of breast cancer resistance protein

**DOI:** 10.1016/j.brainres.2011.11.053

**Published:** 2012-02-03

**Authors:** Christopher P. Watson, Murat Dogruel, Larisa Mihoreanu, David J. Begley, Babette B. Weksler, Pierre O. Couraud, Ignacio A. Romero, Sarah A. Thomas

**Affiliations:** a King's College London, Institute of Pharmaceutical Science, Waterloo, London, UK; b Weill Medical College of Cornell University, New York, NY, USA; c INSERM, U1016, Institut Cochin, Paris, France; d Cnrs, UMR8104, Paris, France; e Univ Paris Descartes, Paris, France; f The Open University, Department of Life Sciences, Walton Hall, Milton Keynes, UK

**Keywords:** BBB, blood–brain barrier, BCRP, breast cancer resistance protein, CT, combination therapy, HAT, human African trypanosomiasis, NECT, nifurtimox–eflornithine combination therapy, S1, stage 1 of human African trypanosomiasis, S2, stage 2 of human African trypanosomiasis, Human African trypanosomiasis, Blood–brain barrier, Nifurtimox, Eflornithine, Breast cancer resistance protein

## Abstract

Human African trypanosomiasis (HAT) is a parasitic disease affecting sub-Saharan Africa. The parasites are able to traverse the blood–brain barrier (BBB), which marks stage 2 (S2) of the disease. Delivery of anti-parasitic drugs across the BBB is key to treating S2 effectively and the difficulty in achieving this goal is likely to be a reason why some drugs require highly intensive treatment regimes to be effective. This study aimed to investigate not only the drug transport mechanisms utilised by nifurtimox at the BBB, but also the impact of nifurtimox–eflornithine combination therapy (NECT) and other anti-HAT drug combination therapies (CTs) on radiolabelled-nifurtimox delivery in an *in vitro* model of drug accumulation and the human BBB, the hCMEC/D3 cell line. We found that nifurtimox appeared to use several membrane transporters, in particular breast-cancer resistance protein (BCRP), to exit the BBB cells. The addition of eflornithine caused no change in the accumulation of nifurtimox, nor did the addition of clinically relevant doses of the other anti-HAT drugs suramin, nifurtimox or melarsoprol, but a significant increase was observed with the addition of pentamidine. The results provide evidence that anti-HAT drugs are interacting with membrane transporters at the human BBB and suggest that combination with known transport inhibitors could potentially improve their efficacy.

## Highlights

► A human *in vitro* blood–brain barrier model used to elucidate nifurtimox transport. ► Nifurtimox was found to be a substrate for the transport protein BCRP. ► The transporter P-gp was not involved in nifurtimox transport. ► Pentamidine caused the accumulation of nifurtimox to increase in the model.

## 1 Introduction

Human African Trypanosomiasis (HAT) is caused by *Trypanosoma brucei gambiense* (*T. b. gambiense*) and *Trypanosoma brucei rhodesiense* (*T. b. rhodesiense*), two species of parasitic protozoans belonging to the genus *Trypanosoma*. The trypanosomes are spread by the biting Tsetse fly which acts as an intermediate host. The disease, if left untreated, then manifests as two distinct stages. The first stage (S1) is generally asymptomatic and characterized by presence of the parasites in the blood and lymphatic systems of the human host. The second stage (S2) is characterized by parasites in the brain and cerebrospinal fluid (CSF) and can occur months (*T. b. rhodesiense*) or years *(T. b. gambiense*) after the initial infection. In S2, a variety of central nervous system (CNS) disorders become apparent including insomnia and changes in sleeping cycle which give the disease the name ‘sleeping sickness’ (for a recent review of HAT's effects on the CNS see Kristensson et al., 2010). If HAT remains untreated it is fatal, thus anti-parasitic chemotherapy is crucial. Fortunately, several drugs are available to treat the disease but have different efficacies depending on the disease stage and pathogen being targeted. The drugs also pose several other problems; they can be expensive, require intensive administration programmes which are unrealistic in a resource poor setting and some are toxic to patients. Treatment of S2 requires that the drug crosses the blood–brain barrier (BBB); the highly specialised microvasculature that separates the cerebral tissue from the blood circulation (Abbott et al., 2006). S1 acting drugs are pentamidine and suramin which are effective against *T. b. gambiense* and *T. b. rhodesiense,* respectively (Brun et al., 2010; Sanderson et al., 2007; Sands et al., 1985). S2 drugs are melarsoprol, eflornithine and nifurtimox. Several recent reviews discuss the S2 acting drugs in further detail (Brun et al., 2010; Lutje et al., 2010).

Our research group has investigated the ability of suramin, pentamidine, eflornithine and nifurtimox to cross the BBB using an *in situ* brain/choroid plexus perfusion technique in anaesthetised mice (Jeganathan et al., 2011; Sanderson et al., 2007, 2008, 2009). Our latest study focused on nifurtimox, an anti-parasitic nitrofuran that was originally used to treat Chagas disease; a closely related condition to HAT caused by *Trypanosoma cruzi* (Gonnert and Bock, 1972; Haberkorn and Gonnert, 1972), but has since been used in compassionate treatment for HAT when other methods have failed (Moens et al., 1984; Van Nieuwenhove, 1992). Nifurtimox is now used against S2 in combination with eflornithine (Checchi et al., 2007). Nifurtimox is cheap, orally active and effective against *T. b. gambiense* and, to a lesser extent, *T. b. rhodesiense* (Bouteille et al., 2003; Haberkorn, 1979; Lutje et al., 2010). Importantly, our group have shown that nifurtimox is able to cross the murine BBB *in situ*, but undergoes an efflux removal process from the brain via an unidentified process, in which the adenosine triphosphate (ATP) binding cassette (ABC) transporter P-glycoprotein, (P-gp) is not involved (Jeganathan et al., 2011). The identify of this efflux mechanism is of special interest with the fact that nifurtimox–eflornithine combination therapy (NECT) is now becoming the first course of treatment against S2 HAT (Yun et al., 2010), having been shown to both improve efficacy and reduce harmful side effects (Priotto et al., 2007, 2009). The precise mechanisms behind the success of this particular combination therapy (CT) have yet to be fully revealed, however, it is possible CT could improve delivery to the brain. Our group have shown that nifurtimox delivery to the mouse brain is improved with the addition of the S1 acting drug pentamidine (Jeganathan et al., 2011), which we have previously identified as being a substrate for cellular transport mechanisms at the BBB, including P-gp (Sanderson et al., 2009). These findings highlight not only the need to elucidate the transport mechanisms utilized by nifurtimox at the BBB, but also the effect of CT on its delivery.

Our earlier work has focused on *in vivo* murine models of the BBB, however, in order to translate the research to the human situation this present study uses a human *in vitro* BBB model, the hCMEC/D3 cell line. The hCMEC/D3 cell line is the most promising immortalized human BBB cell line available today, exhibiting many of the characteristics that are essential for a good predictive BBB *in vitro* model (Poller et al., 2008; Weksler et al., 2005). These include expression of tight junction proteins, polarized expression of multiple ABC/SLC transporters and restrictive permeability (Dauchy et al., 2009; Tai et al., 2009b). The following study is the first to investigate nifurtimox transport interactions in a human model of the BBB.

## 2 Results

### 2.1 hCMEC/D3 — expression of endothelial cell marker von Willebrand factor

**Fig. 1:**
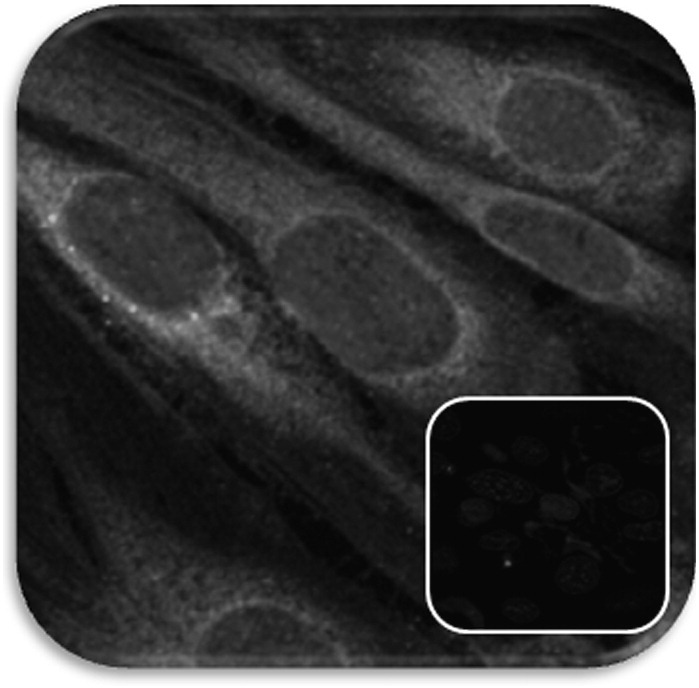
Immunofluoresence of endothelial cell marker vWF in hCMEC/D3 cells. vWF was stained in 4% formaldehyde fixed hCMEC/D3 cells grown on rat-tail collagen type-1 coverslips, as described in Section 4.3 of the Experimental procedures. Cell nuclei were counterstained with 1 μg/ml DAPI. For negative staining, fluorescent secondary antibodies alone were incubated with the cells, along with DAPI (inset). Viewed 63 × with oil emersion with a Zeiss LSM700 confocal microscope and images analysed with Zen 2009 software.

We confirmed the endothelial cell phenotype by staining monolayers of cells grown on collagen-coated coverslips for vascular endothelial marker, von Willebrand factor (vWF) (Fig. 1).

### 2.2 Influence of self-inhibition on [^3^H]nifurtimox accumulation

**Fig. 2:**
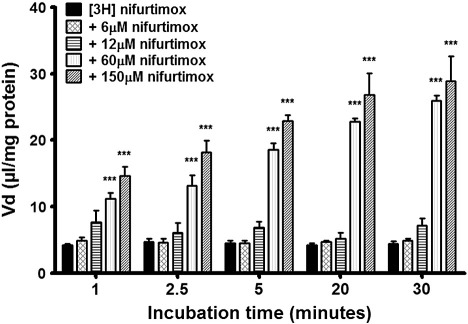
Influence of self-inhibition on [^3^H]nifurtimox accumulation. Unlabelled nifurtimox was added to accumulation buffer in the ranges of 6 μM–150 μM in 0.05% DMSO to view the impact of self-inhibition on the accumulation of [^3^H]nifurtimox in confluent monolayers of hCMEC/D3 cells in 96 well plates. At concentrations of 6 μM and 12 μM, unlabelled nifurtimox caused no change in accumulation of [^3^H]nifurtimox, but at concentrations of 60 μM and 150 μM, a highly significant increase in [^3^H]nifurtimox accumulation compared to controls was observed at all time points (***p < 0.001). All data expressed as mean ± S.E.M, n = 4–6 (plates), with 6 replicates (wells) per plate Data were analyzed with SigmaPlot 11.0.

By varying the concentrations of unlabelled nifurtimox in accumulation buffer alongside [^3^H]nifurtimox and [^14^C]sucrose, we were able to assess any roles played by major BBB transport proteins in the transport and subsequent accumulation of [^3^H]nifurtimox and [^14^C]sucrose, compared to appropriate controls. Accumulation of [^3^H]nifurtimox was not significantly affected by the addition of unlabelled nifurtimox at a clinically relevant dose of 6 μM or an increased dose of 12 μM (Fig. 2). The addition of 60 μM and 150 μM unlabelled nifurtimox, however, caused significant increases in [^3^H]nifurtimox accumulation at all time points (*p < 0.001*) compared to DMSO [^3^H]nifurtimox controls.

### 2.3 Roles of P-gp and BCRP in [^3^H]nifurtimox accumulation

**Fig. 3:**
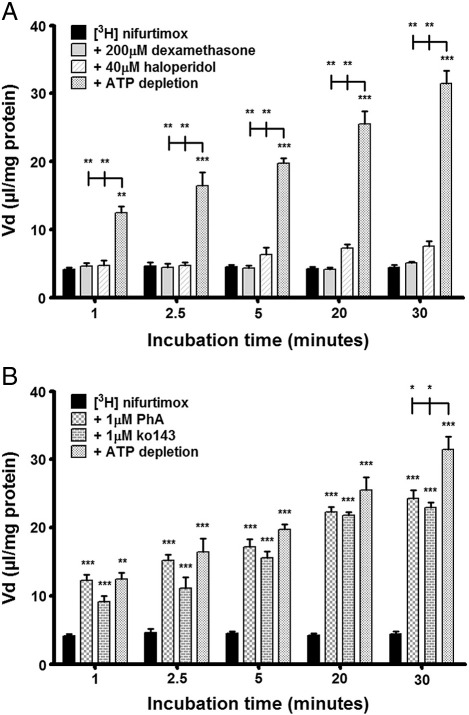
The effects of P-gp and BCRP on [^3^H]nifurtimox accumulation. To assess the roles played by the ABC transporters, P-gp and BCRP, in the transport of nifurtimox, the accumulation of [^3^H]nifurtimox in confluent monolayers of hCMEC/D3 cells in 96 well plates was observed, with or without known transporter interacting drugs, in the presence of 0.05% DMSO. The P-gp substrate and inhibitor (dexamethasone and haloperidol respectively) caused no significant alteration in the accumulation of [^3^H]nifurtimox. ATP depletion caused a significant increase at all time points compared to both control data and P-gp inhibition (*A*). PhA and ko143 (a BCRP substrate and BCRP inhibitor respectively), caused a significant increase in [^3^H]nifurtimox accumulation in the cells compared to control data (***p < 0.001). The values were comparable to those generated by ATP depletion which also caused a significant increase compared to controls at all time points (**p < 0.01, ***p < 0.001) until the 30 minute time point when ATP depletion caused a significant increase in accumulation compared to both inhibitors (*p < 0.05) (*B*). All data expressed as mean ± S.E.M, n = 4–6 (plates), with 6 replicates (wells) per plate. Data were analyzed with SigmaPlot 11.0.

To assess any roles played by major BBB transport proteins in the transport and subsequent accumulation of [^3^H]nifurtimox and [^14^C]sucrose, a variety of drugs were used individually in the accumulation buffer alongside [^3^H]nifurtimox and [^14^C]sucrose and compared to appropriate controls. The influences of P-gp and BCRP in the transport of [^3^H]nifurtimox, were tested using four drugs that have previously been shown to decrease the functions of these transport proteins (Table 1). For P-gp assessment we used haloperidol (40 μM) and dexamethasone (200 μM) and for BCRP, ko143 (1 μM) and pheophorbide A (PhA) (1 μM). The results showed that the P-gp acting drugs, haloperidol and dexamethasone, had no affect on [^3^H]nifurtimox accumulation (Fig. 3A), whereas significant increases in [^3^H]nifurtimox accumulation were observed with the addition of both the BCRP acting drugs, ko143 and PhA (both *p < 0.001* inhibitor against controls) (Fig. 3B).

To further assess roles played by ABC transporters in [^3^H]nifurtimox accumulation, cellular ATP was depleted using 10 mM 2-deoxy-d-glucose (2-DG, see Experimental procedures Section 4.5). This resulted in a 76% depletion of intracellular ATP compared to untreated controls (data not shown). This effectively increased the accumulation of [^3^H]nifurtimox in the cells compared to controls at all time points. When comparing the effect of ATP depletion to that of inhibiting P-gp transport (Fig. 3A), there was a significant difference with ATP depletion causing an increased [^3^H]nifurtimox accumulation when compared with P-gp inhibition(*p < 0.01*). In contrast, comparing the effect of ATP depletion to that of BCRP inhibition (Fig. 3B) showed that these two treatments caused similar changes to [^3^H]nifurtimox accumulation after 1, 2.5, 5 and 20 min, although it was noted that after 30 minutes ATP depletion caused a significantly greater increase (by 17–20%) in [^3^H]nifurtimox accumulation (*p < 0.05)*. There were no significant differences in [^14^C]sucrose accumulation between any treatments (data not shown).

### 2.4 Roles of MRP, OATPs and OATs in [^3^H]nifurtimox accumulation

**Fig. 4:**
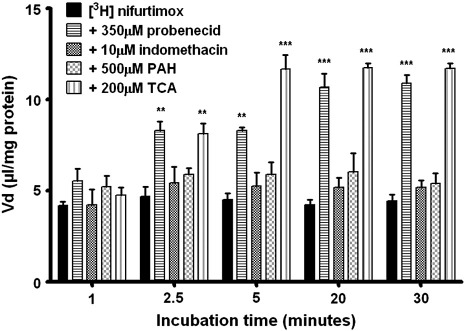
The effects of OATs, OATPs and MRPs on [^3^H]nifurtimox accumulation. To assess the roles played by members of the transport families OATs, OATPs and MRPs in the transport of nifurtimox, the accumulation of [^3^H]nifurtimox in confluent monolayers of hCMEC/D3 cells in 96 well plates was observed, with or without known transporter interacting drugs, in the presence of 0.05% DMSO. The OAT interacting drug, PAH and the MRP1 interacting drug, indomethacin, caused no significant changes in the accumulation of [^3^H]nifurtimox. From 2.5 minutes onwards, both probenecid (OATs, OATPs and MRPs) and TCA (OATPs) caused significant increases in the accumulation (**p < 0.01, ***p < 0.001). Data were analyzed with SigmaPlot 11.0. All data expressed as means ± S.E.M, n = 4–6 (plates), with 6 replicates (wells) per plate. Data were analyzed with SigmaPlot 11.0.

Probenecid (350 μM) was used to assess any initial contributions to [^3^H]nifurtimox and [^14^C]sucrose accumulation from proteins separate to P-gp and BCRP; namely multi-drug resistance associated proteins (MRP) 1 and 2, organic anion-transporting polypeptides (OATPs) and organic anion transporters (OATs) (Table 1). Fig. 4 illustrates the time dependent effect of probenecid on [^3^H]nifurtimox accumulation. This was not matched by the presence of 10 μM indomethacin, where no significant change to [^3^H]nifurtimox was observed at any time point. Taurocholic acid (TCA, 200 μM) and para-aminohippuric acid (PAH, 500 μM) were then used to assess function of OATPs and OATs respectively. The addition of TCA caused significant changes in [^3^H]nifurtimox accumulation from 2.5 min (*p < 0.01*) and onwards when all three time-points showed significant increases (*p < 0.001* Fig. 3), albeit less than those observed with the BCRP inhibitors. PAH caused no significant differences in accumulation of [^3^H]nifurtimox at any time point. No significant differences in [^14^C]sucrose accumulation between any treatments were observed (data not shown).

### 2.5 Combination therapy and [^3^H]nifurtimox accumulation

**Fig. 5:**
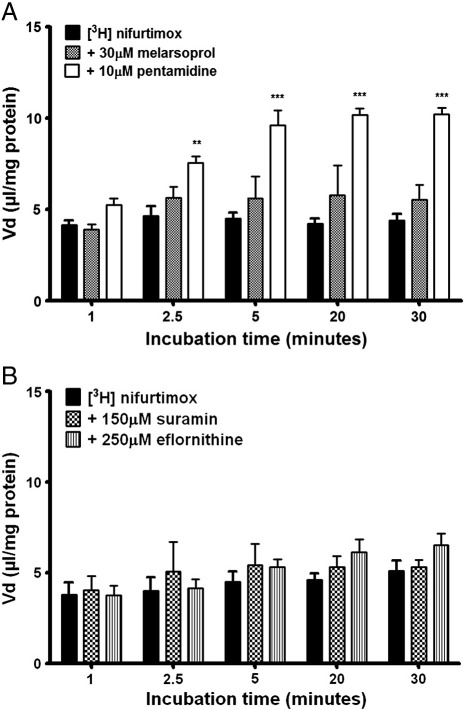
Combination therapy and its effect on accumulation of [^3^H]nifurtimox. Other anti-HAT drugs were added with [^3^H]nifurtimox in the accumulation buffer to assess their impact on accumulation. Unlabelled melarsoprol caused no significant change, but pentamidine induced a significant increase in accumulation of [^3^H]nifurtimox, from 2.5 minutes onwards (**p < 0.01, ***p < 0.001) (*A*, DMSO present). Neither unlabelled suramin nor eflornithine caused a significant change in the accumulation of [^3^H]nifurtimox (*B,* DMSO absent). Data were analyzed with SigmaPlot 11.0. All data expressed as means ± S.E.M, n = 6 (plates) per graph, with 6 replicates (wells) per plate. Data were analyzed with SigmaPlot 11.0.

With CTs becoming the treatments of choice for HAT, the effect of their addition to the accumulation buffer was observed on [^3^H]nifurtimox and [^14^C]sucrose accumulation. The accumulation of [^3^H]nifurtimox in the hCMEC/D3s was not significantly affected by unlabelled melarsoprol (30 μM), whereas unlabelled pentamidine (10 μM) caused an increase at 2.5 min (*p < 0.01*) and this was maintained onwards to 30 min (*p < 0.001*), in comparison to DMSO controls (Fig. 5A).

The effect of eflornithine (250 μM) and suramin (150 μM) on the accumulation of [^3^H]nifurtimox (without the presence of DMSO) saw no significant changes arise (Fig. 5B). There were no significant differences in [^14^C]sucrose accumulation between any of these treatments, or between DMSO and no DMSO controls (both [^3^H]nifurtimox and [^14^C]sucrose, data not shown).

### 2.6 Cytotoxicity of compounds used

**Fig. 6:**
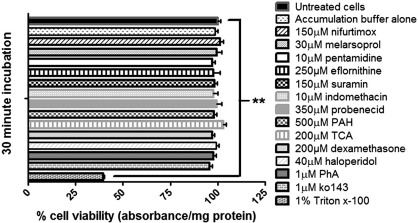
Cytotoxic effects of compounds used in the study. The test compounds were assessed for any cytotoxic effects using an MTT assay during 30 minute incubations with confluent monolayers of hCMEC/D3 cells in 96 well plates, as described in Section 4.7 of Experimental procedures. The results are expressed as percentage viability ± S.E.M and compared to control untreated cells, which were incubated for 30 min in normal medium (Section 4.2). None of the compounds induced a significant change in cell viability, except for the positive control, 1% Triton x-100 (**p < 0.01). Data were analyzed with SigmaPlot 11.0 with n = 3 (plates), 4 replicates per n (4 wells).

The potential of the compounds used in this study to cause cytotoxicity was assessed using an MTT assay and the effect compared to untreated control endothelial cells (hCMEC/D3) (Fig. 6). There were no significant differences on cell viability after 30 minutes exposure to the drugs, except when using the positive control 1% Triton X-100 (*p < 0.01*).

### 2.7 BCRP and P-gp protein expression in hCMEC/D3s

**Fig. 7:**
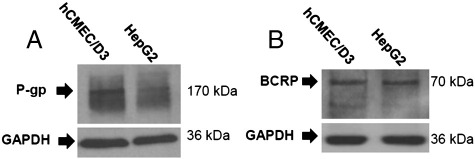
Expression of P-gp and BCRP in the hCMEC/D3 cells. Confluent monolayers of hCMEC/D3s and flasks of HepG2 cells were lysed in TGN buffer and the expression of P-gp (*A*) and BCRP (*B*) was analysed using western blotting, as described in Section 4.8 of the Experimental procedure. GAPDH was stained as a loading control.

Mouse anti-human BCRP/ABCG2 and mouse anti-human P-gp/MDR1 monoclonal antibodies were used to detect protein expression of BCRP and P-gp in this cell system using SDS-PAGE and Western blotting. Bands detected at the expected molecular weights of 70 kDa for BCRP and 170 kDa for P-gp confirmed their expression using SDS-PAGE and Western blot analysis (Figs. 7A and B). HepG2 cell lysates were used as positive controls (Vander Borght et al., 2008; Wojtal et al., 2006).

## 3 Discussion

Human African trypanosomiasis has a huge impact, both social and economic, on affected sub-Saharan communities. It requires constant surveillance and careful implementation of preventative measures by the authorities to successfully combat the disease. Collapses of disease surveillance and changes in political agenda have allowed HAT's prevalence to increase and this is one of the reasons the disease has not been eradicated. Another reason is due to the unsatisfactory treatment of the disease due to the fact that the anti-HAT drugs available are expensive, can be extremely difficult to successfully administer, have limited efficacy and can cause severe adverse reactions. These features combined with a lack of understanding about anti-HAT drugs highlight the need for more research into the treatment of this disease.

The aim of this study was to investigate whether BBB transport proteins were being utilized by the emerging drug of choice for treating HAT, nifurtimox, and also investigated the effects, if any, of anti-HAT CT on its delivery. We used the hCMEC/D3 cell line as an *in vitro* model of the human BBB, first confirming an endothelium phenotype through staining for vWF. We then investigated the effect of unlabelled nifurtimox on [^3^H[nifurtimox accumulation and whilst the lower concentrations (6 and 12 μM) caused no significant change, the higher concentrations (60 μM and 150 μM) saw a large increase in [^3^H]nifurtimox accumulation illustrating that nifurtimox is a substrate for an efflux transporter in this human BBB model. Our group has previously shown that nifurtimox is a substrate for an efflux protein at the murine BBB, which is unlikely to be P-gp, as shown by the use of P-gp deficient animals (Jeganathan et al., 2011). P-gp is expressed at the luminal membrane of the human BBB and removes a wide variety of substrates from the endothelial cell cytoplasm. The lack of interaction between nifurtimox and P-gp was also evident in the hCMEC/D3s through the use of the P-gp substrate, dexamethasone, and the specific P-gp inhibitor (at 40 μM), haloperidol, which did not cause any significant differences in [^3^H]nifurtimox accumulation over the 30 minute incubation period. However, a promising potential efflux transporter for nifurtimox was suggested in our earlier animal study (Jeganathan et al., 2011). Further investigation in the hCMEC/D3s confirmed this efflux transporter to be BCRP with both the BCRP substrate, PhA, and the BCRP specific inhibitor (used in the range of 0.1–1 μM), ko143, causing large increases in [^3^H]nifurtimox accumulation. Importantly, the increase in [^3^H]nifurtimox accumulation caused by these BCRP modulators is in line with that seen with 60–120 μM unlabelled nifurtimox. BCRP, like P-gp, is expressed luminally at the BBB and both these proteins are members of the ABC transporter superfamily which play key physiological roles in protecting tissues from toxic xenobiotics and other potentially harmful endogenous metabolites. ABC transporters require energy in the form of ATP to pump drugs out of the brain against concentration gradients. This ABC-transporter dependence on ATP was exploited here when we depleted cellular ATP by inhibiting glycolysis using the well established inhibitor 2-DG (Wang et al., 2011; Whiteman et al., 2002). ATP depletion resulted in accumulation values comparable to those generated with BCRP inhibitors but not with P-gp inhibitors. At the 30 minute stage, accumulation of [^3^H]nifurtimox using BCRP inhibitors was approximately 83% of the accumulation produced by ATP depletion. These increases in [^3^H]nifurtimox accumulation induced by ATP depletion further supports the evidence that P-gp does not have a role in nifurtimox transport, but BCRP plays a crucial one. The protein expression of both P-gp and BCRP was confirmed in the hCMEC/D3s by Western blot, which is consistent with the findings of several other groups (Poller et al., 2008; Tai et al., 2009a; Weksler et al., 2005). These data suggest that not only is BCRP functional in the hCMEC/D3s but perhaps inhibiting BCRP could improve the delivery and efficacy of nifurtimox. Indeed, that nifurtimox could be a substrate for BCRP that has been previously indicated (Garcia-Bournissen et al., 2010; Jeganathan et al., 2011). In their study investigating nifurtimox transfer in breast milk, Garcia-Bournissen et al*.* suggested that as the antibiotic, nitrofurantoin, is structurally related to nifurtimox and is a substrate for BCRP (Merino et al., 2005), perhaps nifurtimox may also be a substrate (Garcia-Bournissen et al., 2010). The findings of our study provide direct evidence of this hypothesis for the first time in a human *in vitro* BBB model.

To further investigate the roles of other transport systems with nifurtimox, a variety of other drugs were used to affect transport activity of MRPs, OATs and/or OATPs. MRPs, other members of the ABC transporter superfamily that also mediate brain-to-blood efflux, play important roles *in vivo* to protect the brain from xenobiotics. OATs and OATPs are membrane transport proteins that play large roles in the transport of endogenous molecules across cell membranes. MRP1 expression has previously been shown in the hCMEC/D3s at mRNA (Carl et al., 2010) and protein levels (Weksler et al., 2005). The expression of MRPs 2,3,4 and 5, OATP1, OATPD and OATP2A1 has been shown at mRNA level only in the hCMEC/D3s (Carl et al., 2010; Poller et al., 2008), and they are also expressed in the human brain (Gibbs and Thomas, 2002). However, to date neither protein nor mRNA expression of the OAT superfamily in the hCMEC/D3 cell line has been demonstrated. The data here comply with this knowledge somewhat, suggesting that nifurtimox appears to be a substrate of functional OATP systems, but OATs and MRP1 appear not to be involved. This was demonstrated by use of the broad spectrum MRP, OAT and OATP inhibitor, probenecid; the MRP1 inhibitor, indomethacin, the OATP competitive inhibitor; TCA and the OAT competitive inhibitor, PAH. OATPs are bidirectional drug transporters (Nozawa et al., 2005) and the increases in accumulation with the addition of TCA could provide some evidence for their role in nifurtimox transport. However, although indomethacin is commonly used as a MRP1 inhibitor, it has also been shown to be a substrate of OATPs and OATs, albeit at different concentration ranges than used here (Parepally et al., 2006). Notably the changes in [^3^H]nifurtimox accumulation were much smaller than those seen with the BCRP specific drugs and accumulation following ATP depletion, suggesting only minor roles for OATPs in comparison to the role played by BCRP. It is also important to point out that some groups have found that probenecid is a BCRP substrate *in vitro,* and this is also a possible explanation for the increase in [^3^H]nifurtimox accumulation using this drug (Merino et al., 2006). However, other groups have found no such evidence of BCRP/probenecid interaction (Pan et al., 2007). It has also been reported that neither TCA (Suzuki et al., 2003) nor indomethacin (Elahian et al., 2010) interacts with BCRP. The concentrations of drugs used in this study were carefully chosen to follow those used in previous *in vivo* studies by our group, to be in line with the clinically relevant doses used in the field (with the anti-HAT drugs) and to be in line with those reported in publications such as Matsson et al. 2009. These data and findings by other groups highlight that drugs affecting transport activity must be used at specific concentrations to affect the targeted transport proteins.

With combination therapy becoming the most promising method of clinical S2 HAT treatment, we also investigated whether other unlabelled anti-HAT drugs could modulate the accumulation of [^3^H]nifurtimox in human brain endothelium. In line with previous work by our group, an increase in [^3^H]nifurtimox accumulation was seen with the addition of 10 μM pentamidine. Pentamidine, a S1 acting drug, has been previously shown by our group to be a substrate for P-gp and is also transported by other transport proteins including MRP. This present study with P-gp inhibitors suggests that perhaps nifurtimox also interacts with other membrane transporters. BCRP and members of the OATPs could be candidates as indicated by their interactions with PhA, ko143, probenecid and PAH. However, no other drugs from the CT experiments, including eflornithine, caused any significant changes in [^3^H]nifurtimox accumulation in the cells, which contradicts our previous findings that eflornithine, suramin and melarsoprol caused decreases in the brain distribution of [^3^H]nifurtimox *in vivo* (Jeganathan et al., 2011). One must of course be careful when comparing *in vivo* brain distribution and *in vitro* endothelial cell accumulation data. When observing the *in vivo* BBB endothelial cell pellet analysis for [^3^H]pentamidine previously published by our group, it was evident that the drug accumulated in the cells (Sanderson et al., 2009). [^3^H]nifurtimox also accumulated *in vivo* in BBB endothelial cell pellets, and the effect on accumulation with CT were similar to those reported here with an increase observed with the addition of unlabelled pentamidine and little or no difference with the other drugs (Jeganathan et al., 2011). The reasoning behind the improved cure rates of patients using NECT compared to eflornithine alone, based on our results, is unlikely to be due to the interactions of the drugs with membrane transporters at the level of the brain capillary endothelium. It has been stipulated that the arrestment of parasite defences caused by eflornithine allows the efficacy of nifurtimox to be improved and perhaps this is the main reason behind NECT success (Priotto et al., 2009).

The mechanism by which nifurtimox enters the cells remains unknown. It is likely that the lipophilic properties of nifurtimox (with an octanol–saline partition coefficient of 5.46 Jeganathan et al., 2011) allow it to cross cell membranes by passive diffusion and previous work has shown that it not only appears to use a transcellular route of entry, but enters the mouse brain at sufficient amounts to be effective in killing trypanosomes (Jeganathan et al., 2011). However, the role played, if any, by blood-to-brain transporters remains elusive. Any effect that the drugs had on the expression of transporters in the hCMEC/D3 cell line has not been assessed here. It has been shown previously that some drugs can upregulate functional expression of drug transporters such as P-gp, and this is well documented with dexamethasone (Narang et al., 2008), but the 30 minute time frames of the experiments in this report were unlikely to be sufficient at inducing any significant increase in expression or activity.

Studying nifurtimox entry and exit to the brain is crucial to improving treatment of second stage HAT, especially now that NECT is fast becoming the treatment of choice. Considering the current usage of NECT, it is somewhat surprising that very little is known about the mechanisms being used by these drugs to gain entry to the human brain. We report here that nifurtimox is a substrate of BCRP and possibly, to a lesser extent, members of the OATP transport family, in an *in vitro* model of the BBB. We also report that, with regards to the combination therapy approach to treating HAT, the combination of clinically relevant doses of eflornithine and nifurtimox does not appear to hamper entry of nifurtimox into the BBB and the reasoning behind its success in the field is unlikely due to transporter interactions at the BBB. In addition, our data suggests that pentamidine could actually improve the delivery of nifurtimox, which is in line with previous work by our group in an animal model.

## 4 Experimental procedure

### 4.1 Materials

Nifurtimox (MW 287.30) was custom labelled with tritium (^3^H 3,4 furam ring) specific activity: 2 Ci/mmol) by Moravek Biochemicals (California, USA). [^14^C]sucrose (4980 mCi/mmol) was purchased from Moravek Biochemicals. Unlabelled suramin, eflornithine and pentamidine isethionate sodium salt were purchased from Sigma Chemical Company (Dorset, UK). Unlabelled nifurtimox and melarsoprol were a kind gift from Professor S. Croft (London School of Hygiene and Tropical Medicine, UK). Probenecid, indomethacin and dimethyl sulfoxide (DMSO) were purchased from Sigma Chemical Company. Dexamethasone and Pheophorbide A (PhA) were purchased from Acros Organics, (Fisher Scientific, Loughborough, UK). Para-aminohippuric acid (PAH) and taurocholic acid (TCA) were purchased from MP Biochemicals, UK. Ko143 and haloperidol were purchased from Tocris Bioscience (Bristol, UK) and Sigma, respectively. The hCMEC/D3 cell line was obtained from Professor Pierre O. Couraud (Institut Cochin, Université Paris Descartes, CNRS, Paris, France) and Dr Ignacio Romero (The Open University, Department of Life Sciences, Walton Hall, Milton Keynes, UK). The EGM-2MV BulletKit was purchased from Lonza (Basel, Switzerland). All cultureware was Nunclon brand and purchased from Thermo Scientific, UK. Rat tail collagen 1 and penicillin-streptomycin were purchased from Gibco, Invitrogen, (Paisley, UK). HEPES 1M was purchased from Sigma Chemical Company. Primary mouse anti-P-gp/MDR1 [C219] (ab3364), anti-BCRP/ABCG2 [BXP-21] (ab3380) and mouse anti-GAPDH monoclonal antibodies [6C5] (ab8245), rabbit polyclonal secondary antibody (HRP) (ab6728) were purchased from Abcam, Cambridge, UK. Goat anti-rabbit Alexa Fluor 488 was purchased from Invitrogen, UK. HepG2 cells were a kind gift from Mr Enrico Cristante (Imperial College London, UK). Rabbit anti-human von Williebrand factor (vWF) (P0226, Dako, Stockport, UK) was a kind gift from Dr Sarah Chapple (King's College London).

### 4.2 Cell culture

The hCMEC/D3s were cultured in EBM-2 endothelial growth medium supplemented with HEPES, penicillin–streptomycin, 2.5% foetal bovine serum (FBS), insulin-like growth factor-1, vascular endothelial growth factor, epidermal growth factor, hydrocortisone and basic fibroblast growth factor from the EGM-2MV BulletKit as previously described (Poller et al., 2008). All cells used in the experiments were seeded at a density of 2.5 × 10^4^ cells/cm^2^ and were between passages 28 and 35. Before seeding, cells were checked for viability by 0.4% Trypan Blue solution in a haemocytometer. Cultureware was coated with 0.1 mg/ml rat tail collagen type 1 for 2 h at 37 °C prior to seeding. Cells were cultured in an incubator with a saturated humidity at 37 °C in 5% CO_2_ and 95% fresh air and grown to 80–90% confluency before seeding (after 3 days). For experiments, cells were grown to 100% confluency (which was reached at 4 days) in collagen coated 96 well plates and then left for a further 3 days until experiments (7 days after seeding). Medium was changed every 2–3 days. Protein expression (BCA® protein assay, Thermo Scientific, Loughborough, UK) and integrity of plasma membranes ([^14^C]sucrose) were monitored to confirm cell viability and used for correction factors (see experimental details below). HepG2 cells were cultured in 25 cm^2^ flasks in Dulbecco's modified Eagle's medium (DMEM, Gibco, Invitrogen) with 10% FBS (PAA, Yeovil, A15-151).

### 4.3 Staining of vWF

The endothelial phenotype of the hCMEC/D3s was first confirmed by staining for endothelial cell marker vWF (Fig. 1) (Schram et al., 2003). Cells were grown on rat-tail collagen type 1 coated glass coverslips and then fixed using 4% formaldehyde in PBS for 10 min at 4 °C. The coverslips were then washed three times with PBS and treated for 5 min with 0.1% Triton X-100 in PBS at room temperature (RT). Following this permeablisation step, coverslips were washed three times in PBS and then non-specific sites were blocked with PBS containing 10% serum, 0.1% Triton X-100 for 30 min at RT. The coverslips were then incubated overnight at 4 °C with primary antibody (1:200 for rabbit anti-human vWF in PBS). Following overnight incubation, coverslips were washed three times with PBS and goat anti-rabbit Alexa Fluor 488 (1:200 in PBS) was added for 1 h at RT. Following secondary incubation, coverslips were washed in PBS twice, and incubated in PBS containing 1 μg/ml DAPI nucleus stain (New England Biolabs, Bristol, UK) for 30 min at RT. Coverslips were then washed a final time in PBS, dipped in distilled water and mounted onto slides with PVA-DABCO®, before viewing with a Zeiss LSM710 confocal microscope and image analysis software Zen 2009 (Zeiss, Germany).

### 4.4 Drug accumulation assays

Drug accumulation experiments were performed on confluent monolayers of hCMEC/D3s, grown in the centre 60 wells of 96 well plates. Accumulation studies are based on a previous study (Chishty et al., 2004). Medium was removed from wells and replaced with a 200 μl aliquot of [^3^H]nifurtimox (120nM) and [^14^C]sucrose (972 nM) in accumulation buffer (consisting of 135 mM NaCl, 10 mM HEPES, 5.4 mM KCL, 1.5 mM CaCl_2_, 1.2 mM MgCl_2_, 1.1 mM d-glucose, and distilled water, pH 7.4). Columns of wells (6 wells/column, 10 columns/plate) were exposed to the [^3^H]drug/[^14^C]drug/buffer mix at five different time periods (1, 2.5, 5, 20 and 30 min). This allowed assessment of drug accumulation in the cells. The accumulation assays were performed on a temperature-controlled shaker (THERMOstar, BMG labtech, Offenburg, Germany) at 37 °C and 120 rpm. Once each column of cells had been exposed for the correct amount of time, the wells were washed 3 times with ice-cold phosphate buffered saline (1 × PBS, Gibco, Invitrogen, UK) to stop transport processes and remove drugs and buffer that had not accumulated in the cells. The cells were then lysed by adding 200 μl of 1% Triton x-100 (Sigma, UK) per well for 1 h at 37 °C to liberate any accumulated radiolabelled drug and solubilise the cell proteins. 100 μl of each well was then added to scintillation vial along with 4 ml scintillation fluid (Optiphase Hisafe 2, PerkinElmer, UK) added and samples counted as described previously (Sanderson et al., 2008). The remaining 100 μl in each well was used to perform a BCA™ protein assay, using bovine serum albumin as standards, and measured spectrophotometrically on a Labsystems Multiscan reader with Ascent software. Total accumulation of [^3^H]nifurtimox was calculated as the sum of accumulation and efflux and termed the volume of distribution (V_d_). V_d_ is derived from the ratio of dpm/mg protein to dpm/μl buffer. The V_d_ values for [^3^H]nifurtimox were corrected with the V_d_ values for [^14^C]sucrose which is a marker of non-specific binding and extracellular space.

### 4.5 Transporter inhibition assays

To study the transport mechanisms being utilized by nifurtimox, a range of unlabelled nifurtimox concentrations in the presence of 0.05% dimethyl sulfoxide (DMSO) (6 μM, 12 μM, 60 μM and 150 μM) were also used alongside [^3^H]nifurtimox and [^14^C]sucrose in the accumulation buffer to assess the effect on [^3^H]nifurtimox efflux from the cells. We also used a series of established transporter interacting (substrates and inhibitors) drugs were used alongside [^3^H]nifurtimox and [^14^C]sucrose in accumulation buffer. The impact of these drugs on [^3^H]nifurtimox and [^14^C]sucrose accumulation in the cells was assessed at 1, 2.5, 5, 20 and 30 min. Haloperidol (40 μM), ko143 (1 μM), indomethacin (10 μM) pheophorbide A (PhA) (1 μM), taurocholic acid (TCA) (200 μM), para-aminohippuric acid (PAH) (500 μM), dexamethasone (200 μM) or probenecid (350 μM) were added to accumulation buffer in 0.05% DMSO in individual experiments to inhibit different transport systems (Table 1).

To further assess the impact of ABC-transporters on the accumulation of [^3^H]nifurtimox, cells were depleted of ATP by incubating them for 1 h in glucose-free DMEM containing 10 mM 2-deoxy-d-glucose (2-DG, Sigma), and cellular ATP was determined using the Promega Enliten® ATP Assay System kit (Promega, Southampton, UK). Briefly, cells were grown in 24 well plates for 7 days before their medium was removed, washed twice with warm glucose free DMEM (Gibco, Invitrogen) and incubated for 1 h in glucose-free DMEM containing 10 mM 2-DG which is a well documented inhibitor of glycolysis and results in a decrease in intracellular ATP *in vitro* (Wang et al., 2011). After this incubation step, the 2-DG solution was removed and cells were incubated in 100 μl of 2% trichloroacetic acid (TCA, Sigma) in glucose-free DMEM, also containing 0.002% xylenol blue dye (a pH colour indicator, Sigma) at RT for 10 min following the manufacturer's direction. TCA both depletes cellular ATP and inhibits enzymes that degrade ATP (Whiteman et al., 2002). As TCA also inhibits the downstream assay, it was neutralised with Tris-acetate (pH 7.75) to bring the total TCA percentage to 0.1% following the manufacturer's direction. Samples were then taken and added with the reconstituted luciferase/luciferin reagent mix from the kit in a sterile white 96-well plate (Nunc) and the ATP luminescence determined in a Biotek Synergy HT luminometer using KC4 software and compared to control cells not treated with 2-DG. For accumulation, cells were treated with 10 mM 2-DG before incubation with [^3^H]nifurtimox as described above.

### 4.6 Combination therapy accumulation

In a series of experiments to assess the impact of CT on [^3^H]nifurtimox cellular accumulation, the clinically relevant concentrations of melarsoprol (30 μM), pentamidine (10 μM), suramin (150 μM) or eflornithine (250 μM) were added to accumulation buffer. DMSO was used to dissolve melarsoprol and pentamidine to give a final concentration of 0.05% DMSO. Control experiments here also contained 0.05% DMSO. For unlabelled eflornithine and suramin and the appropriate controls, no DMSO was used. There was no significant difference between accumulation of [^3^H]nifurtimox with or without 0.05% DMSO (data not shown).

### 4.7 MTT assay

The cytotoxic effects of the drugs used in this study were assessed on confluent monolayers of cells in 96 well plates using an MTT assay. Cells underwent 30 minute incubations with a 200 μl/well aliquot of each drug in accumulation buffer at the concentrations used in the experiments. After 30 min, the buffer was aspirated and replaced with a 100 μl aliquot of 1 mg/ml MTT (3-(4,5-Dimethylthiazol-2-yl)-2,5-diphenyltetrazolium bromide, Sigma, UK) in DMEM without phenol red (Gibco, Invitrogen, UK). The cells were then incubated for 4 h at 37 °C, the solution removed and replaced with 100 μl propan-2-ol per well, and the absorbance was measured. Absorbance values were corrected by protein content (determined using a BCA assay) and expressed as percentage viability compared to control untreated cells.

### 4.8 SDS-PAGE and Western blot

The expression of P-gp and BCRP by the hCMEC/D3 and HepG2 cell lines was analysed by Western blot using Abcam primary mouse anti-P-gp/MDR1 [C219] (ab3364) and mouse anti-BCRP/ABCG2 [BXP-21] (ab3380) monoclonal antibodies at 1:80 and 1:1000 dilutions in PBS-Tween (PBS-T, PBS with 0.05% Tween 20) with 0.5% BSA, (Sigma) respectively. Mouse anti-GAPDH monoclonal antibody [6C5] (ab8245), was used as a loading control, 1:1000 in PBS-T with 0.5% BSA. Confluent monolayers of hCMEC/D3 cells and flasks of HepG2 cells (positive controls) were lysed in TGN lysis buffer (50 mM Tris, 150 mM NaCl, 10% glycerol, 50 mM glycerophosphate B, 1% Tween-20, 0.2% NP-40, all purchased from Sigma, UK), and 25 μg loaded per lane. For P-gp, a precast 4–20% gradient gel was used (Bio-Rad Europe, 456-1093S). For BCRP, a 10% SDS-PAGE acrylamide/bisacrylamide gel was used. Following electrophoresis, proteins were transferred using semi-dry transfer onto methanol activated Immobilon-P PVDF membranes (0.45 μM pore size, Millipore, Ireland), blocked for 2 h at RT in PBS-T with 5% milk powder and incubated overnight at 4 °C with antibody. Membranes were then washed 3 × with PBS-T and incubated for 1 h at RT with rabbit anti-mouse HRP conjugated secondary antibody 1:2000 in PBS-T (Abcam, ab6728), before visualization with enhanced chemiluminescence (ECL,Thermo Scientific, 32209) in a dark room.

### 4.9 Statistical analysis

Comparisons were made between control plates of cells and differences at the 5% level considered significant. Multiple-time accumulation data were analysed by Two Way Repeated Measures ANOVA tests and Holm–Sidak posthoc tests, MTT assay data were compared against controls using a One Way ANOVA using Sigma Plot version 11.0 software (SPSS Science Software UK Ltd., Birmingham UK). All data are expressed as mean ± SEM, except MTT data which are expressed as percentage viability.

## Conflict of interest

The authors acknowledge that there are no conflicts of interest.

## Figures and Tables

**Table 1:** Transporter interacting drugs used in this study. All molecules were used in the presence of 0.05% DMSO and used at the published concentration ranges where they affect transport activity.

Drug	Concentration	Transporters	Source	Reference
Dexamethasone	200 μM	P-gp	MP Biomedicals, UK	(Mark and Waddell, 2006; Mason et al., 2008; Ueda et al., 1992)
Haloperidol	40 μM	P-gp	Sigma, UK	(Matsson et al., 2009)
Ko143	1 μM	BCRP	Tocris bioscience, UK	(Matsson et al., 2009)
Pheophorbide A	1 μM	BCRP	MP Biomedicals, UK	(Weiss et al., 2007)
Indomethacin	10 μM	MRP1	Sigma, UK	(Rosenbaum et al., 2005)
PAH	500 μM	OATs	Acros organics, UK	(Gibbs and Thomas, 2002; Sauer et al., 2010; Sugiyama et al., 2001)
Probenecid	350 μM	MRP-1, MRP-2, OATs, OATPs	Sigma, UK	(Gibbs and Thomas, 2002; Jorgensen et al., 2007; Potschka et al., 2004)
TCA	200 μM	OATPs	Acros organics, UK	(Gibbs and Thomas, 2002; Sugiyama et al., 2001)

PAH — para-aminohippuric acid, TCA — taurocholic acid.
